# A randomized controlled trial to test the effect of simplified guidance with visuals on comprehension of COVID-19 guidelines and intention to stay home if symptomatic

**DOI:** 10.1186/s12889-021-10787-9

**Published:** 2021-05-10

**Authors:** Natalie Gold, Robin Watson, Dale Weston, Felix Greaves, Richard Amlôt

**Affiliations:** 1 Public Health England Behavioural Insights, Public Health England, Wellington House, 133-155 Waterloo Road, London, SE1 8UG UK; 2 Centre for Philosophy of Natural and Social Science, London School of Economics and Political Science, Houghton Street, London, WC2A 2AE UK; 3 Department of Anthropology, Durham University, Dawson Building, South Road, Durham, DH1 3LE UK; 4 Behavioural Science Team, Emergency Response Department Science and Technology, Health Protection Directorate, Public Health England, Porton Down, Salisbury, Wilts SP4 0JG UK; 5 Department of Primary Care and Public Health, Imperial College London, South Kensington, London, SW7 2AZ UK

**Keywords:** COVID-19, Coronavirus, Guidelines, Simplification, Text cohesion

## Abstract

**Background:**

In the COVID-19 pandemic, it is imperative that people understand and comply with self-isolation guidelines. We tested whether a simplified version of the guidelines and a simplified version with visual aids would affect comprehension and intention to self-isolate during the containment phase of the pandemic in the UK, in March 2020, compared to the standard guidelines.

**Methods:**

We conducted an online, three-armed parallel randomized controlled trial. Participants were English and over 18. The survey software randomized them into conditions; they were blind to condition. The control group read the 7-page standard guidelines (the current version at the time of the trial). The intervention groups were given either a 3-page simplified version, with a summary box on the front page and numbered bullet points, or the same simplified version with pictograms illustrating the points in the box. Primary outcomes were comprehension of the guidelines, as measured by the number of correct answers given to six questions about the content, and the proportion who answered that they would ‘definitely’ stay at home for 7 days if symptomatic.

**Findings:**

Recruitment was from 13 to 16 March 2020, with 1845 participants randomised and all data analysed. The Control group averaged 4.27 correct answers, the Simplified 4.20, and the Simplified + visual aids 4.13, out of a possible total of 6 correct answers. There were no differences in comprehension in the unadjusted models; however, when the model was adjusted for demographic variables, there was lower comprehension in the simplified + visual aids condition than in the control, (ß = − 0.16, *p* = 0.04998). There were no statistically significant differences in intention to stay home: Control was 85%, Simplified 83%, and Simplified + visual aids condition 84%.

**Conclusion:**

Simplified guidance did not improve comprehension compared to the standard guidance issued in the containment phase of the COVID-19 pandemic in March 2020, and simplified guidance with visual aids may even have worsened comprehension. Simplified guidance had no effect on intention to stay home if symptomatic. This trial informed COVID-19 policy and provides insights relevant to guidance production in the acute phase of a major public health emergency.

**Supplementary Information:**

The online version contains supplementary material available at 10.1186/s12889-021-10787-9.

## Introduction

In the current COVID-19 pandemic, it is imperative that people understand and comply with guidelines in order to prevent the spread of disease. Self-isolation is an important part of the strategy against COVID-19 [1, 2]. The United Kingdom (UK) guidelines state that people with symptoms need to self-isolate; at the time of writing, the self-isolation period for symptomatic individuals and for any asymptomatic individuals within the household is 10 days [3]. Perhaps unsurprisingly, compliance with quarantine is higher amongst those who understand what they need to do [4]. At the time of the study, we were in the early containment phase of the pandemic, the guidance and restrictions were new. We wanted to know what is the best way to communicate detailed guidance in a novel pandemic situation.

We need a better understanding of the effect of simplification of text on understanding. Simplification includes breaking the text up with headers, use of bullets, and deletion of extraneous words. There is some evidence that simplification can increase understanding. For instance, more technical messaging led to lower recall and intention to comply with instructions in the 2007 San Diego wildfires [5]. There is also a substantial literature showing that simplification of letters, when used in conjunction with other techniques from behavioural science, can have positive effects on behavior [6, 7]. However, the psychological literature on text comprehension is not unequivocal. A substantial body of evidence suggests that, while simplification of texts may improve understanding on average, simplification has differential effects and may decrease the comprehension of some readers [8, 9]. Nor has text simplification always been successful when used as a part of a bundle of techniques to change behaviour [10].

Therefore, in March 2020, in the early stages of the pandemic, we conducted a randomized trial comparing the full text version of the Stay at Home Guidance for people with confirmed or possible coronavirus infection (COVID-19), to a specially created simplified version and a simplified version with added visual aids, to investigate whether they led to differences in comprehension of the guidelines and intention to stay home if symptomatic.

## Methods

### Study design

This was an online between-subject parallel randomized controlled trial with three arms, run on the Behavioural Insight Team’s Predictiv platform, https://www.bi.team/bi-ventures/predictiv/. Participants were randomized using computerized random-number generation to see one of three different versions of the Stay at Home Guidance for people with confirmed or possible coronavirus infection (COVID-19): the full version, a simplified version, or a simplified version with added visual aids. The full text of all three versions is included in the supplementary materials.

### Outcome measures

Our primary outcomes were the participants’ comprehension of the guidance and their intention to stay at home if they had a confirmed COVID-19 diagnosis or symptoms of COVID-19.

We measured comprehension by counting the number of correct answers participants gave to six questions about the content of the guidance, giving a score between 0 and 6. For questions which had multiple correct answers, a point was awarded if they responded with all the correct answers and no incorrect answers. Intention to stay home was coded as a binary variable, with a participant was assigned a 1 if they answered, “definitely stay at home for 7 days” and a 0 otherwise. (The other options were: “I would try to stay home for at least 7 days”, “I definitely **would not** stay home for at least 7 days”, and “Not sure”.) This binary coding was chosen as the guidance at the time was unequivocal about the need for seven-day self-isolation for individuals with a confirmed or possible COVID-19 infection, and so any level of compliance below this would represent non-compliance.

Our secondary outcomes were simplicity, anxiety, and reading time. Ratings of the simplicity of the guidance was an ordinal variable from 1 (not at all easy to understand) to 5 (extremely easy to understand). Participants’ ratings of how anxious the guidance made them feel was also an ordinal measure from 1 (not at all) to 5 (extremely). We recorded the amount of time participants spent reading the guidance in seconds. Since the control and the intervention guidance were different lengths, we also calculated reading time per word, by dividing time by the number of words in each condition (Control = 2465, Simplified = 841, Simplified + visual aids = 840).

### Ethics

The Behavioural Insights Team work in accordance with the Market Research Society’s Code of Conduct [11]. The Research Support and Governance Office at Public Health England do not require an internal review for commissioned work.

### Participants

Participants were recruited via a number of panel providers, so they were people who had registered with a panel and consented to be contacted for surveys. Participants were required to be English and over 18.

Participants were paid a fixed fee of approximately £1 for their time. (The panel providers managed the payment and they determined the exact payment amount, as well as whether the payment was in currency or in points that could be converted into currency or other rewards.) In addition, in order to ensure participants paid attention to the guidance, they were paid 30p for each correct answer they gave on the six comprehension questions, with a mean incentive payment of £1.26 in addition to the fixed fee. Participants gave consent online before starting the survey.

### Randomization and masking

Randomization was done by computer when participants entered the survey. Each participant was randomly assigned a number between 1 and 3, using computerized random number generation, which determined which of the three arms they put in. Participants were blind to treatment condition.

### Interventions

**Fig. 1:**
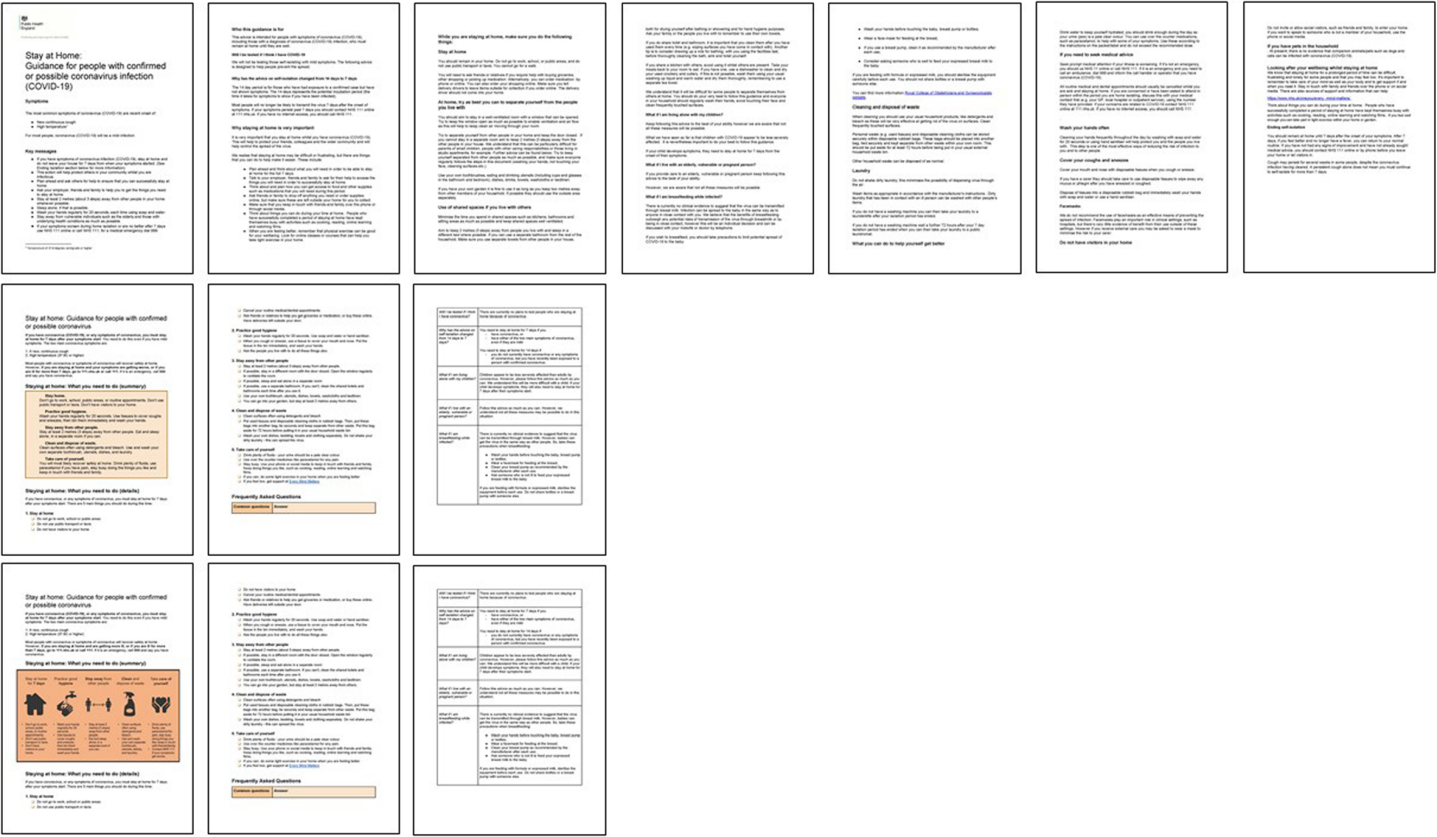
All pages of each guidance condition. Top = control, middle = simplified, bottom = simplified + visual aids

There were three conditions: Standard guidelines (the control), Simplified guidelines, and Simplified guidelines with visual aid. See Fig. 1 for a picture and Appendices 1, 2, 3 for full sized versions.

The standard guidelines were seven pages of text (2468 words). The text started with the symptoms and then a header of ‘Key messages’, which were listed in bullet points and included staying at home for 7 days from when symptoms started, keeping 2 m (3 steps) from other people, washing hands, staying away from the vulnerable, and when to call NHS 111 and 999. This was followed by a section on ‘Who this guidance is for’. On p.3, there was a header ‘Why staying at home is very important’, with a bulleted list of things you can do to make it easier and another header of ‘While you are staying at home, make sure you do the following things’ with the following sub-sections that ran until the end of the guidance: ‘Stay at home’ (p.3), ‘At home, try as best you can to separate yourself from the people you live with’ (p.4), ‘Use of shared spaces if you live with others’ (p.4), which covered children, elderly/ vulnerable/ pregnant, and breast feeding, ‘Cleaning and disposal of waste’ (p.5), ‘Laundry’ (p.6), ‘What you can do to help yourself get better’ (p.6), ‘If you need to seek medical advice’ (p.6), ‘Wash your hands often’ (p.7), ‘Cover your coughs and sneezes (p.7), ‘Facemasks’ (p.7), which at that time were not recommended outside of clinical settings, ‘Do not have visitors in your home’ (p.7), ‘If you have pets in the household’ (p.7), ‘Looking after your wellbeing whilst staying at home’ (p.7), and ‘Ending self-isolation’ (p.8).

The simplified guidelines were three pages of text (1004 words), with orange highlights (136 of the total wordcount was in an orange text box). On the first page, under the title, there were the symptoms and the conditions under which to call NHS 111 or 999, then a text-box summary of what you need to do with the header ‘Staying at home: What you need to do (summary)’. Inside the box, there was a list whose headers were ‘Stay home’, ‘Practice good hygiene’, Stay away from other people’, ‘Clean and dispose of waste’, and ‘Take care of yourself’, with a couple of short sentences with details under each. Then there was a header saying ‘Staying at home: What you need to do (details)’. Going over the second page, the headers from inside the box were repeated as a numbered list, with bullet points under each number with further details. Then, on p.3 was a list of Frequently Asked Questions (FAQs), in a table that had common questions in a column on the left and the answers in a column on the right. The simplified guidelines used orange to highlight the summary box on p.1, in the bullets, and in the header of the FAQ table.

The simplified guidelines with visual aids had the same text as the simplified guidelines, except that the summary box at the beginning displayed the five header messages in a row, with a pictogram for each of the headers and the brief sentences in bullet points underneath. It was 1012 words, of which 135 were in the orange box.

The two simplified guidelines were shorter (3 pages of text instead of 7, with about 40% of the wordcount) and used orange highlights instead of being in black and white. All three versions of the guidelines had the symptoms, the requirement to keep 2 m (3 steps) distance, and the conditions under which one should call NHS 111 on the first page of text (which was p.2. of the control guidance). In addition, the simplified guidelines had information about how to deal with waste and how to take care of yourself in their text box on the first page. This appeared on later pages of the control guidance. The control guidance had more information—some but not all of it was conveyed in the FAQs in the simplified version—and it made more use of paragraphs and a narrative structure, compared to the simplified guidance, which mainly used bullets apart from the FAQs.

### Procedure

Our experiment was conducted on the Behavioural Insight Team’s online experimentation platform Predictiv.^1^ The full materials are in the Appendix. Before starting the survey, participants were shown an information statement and asked if they consented to their data being used for research.

Participants were then randomized into one of three conditions (Standard guidelines, Simplified guidelines, and Simplified guidelines with visual aid) and viewed the relevant version of the guidelines. After viewing the guidelines, participants answered a series of questions.

### Primary outcomes

1. Comprehension of the guidelines:

We asked six comprehension questions:

(i) What should you do if you have coronavirus or symptoms of coronavirus? [Multiple choice from the following: Stay at home and do not leave your house for 7 days after your symptoms started; Visit a GP or hospital; Tell your Local Authority that you have coronavirus; Ring 999 and tell them you have coronavirus]
(ii) If you have to stay at home because you have coronavirus or symptoms of coronavirus, how far away should you stay from other people in your home? [numeric answers in metres or steps, correct answer was 2 m or 3 steps]
(iii) For how many seconds should you wash your hands with soap and water? [numeric answer in seconds, correct answer was 20s]
(iv) If you have to stay at home because you have coronavirus or symptoms of coronavirus, and then you become more sick and need medical help or advice, what should you do? [Multiple choice from the following: Contact NHS 111, or 999 in an emergency; Visit a GP, or for an emergency go to a hospital; Search online for more information; Ask someone to come to your house to help you; Arrange an appointment with your GP]
(v) If you are staying at home because you have coronavirus or symptoms of coronavirus, which of these things should you do? [Tick all that apply : Ask other people to help you get things you need; Have food and groceries delivered to you; Use disinfectant or household cleaner to regularly clean surfaces; Stay away from other people, especially older people; Contact NHS 111 to help you get the things you need to stay at home; Wash your towels and bedsheets everyday; Go to the pharmacy to get medicine if you are in pain; Allow a maximum of three visitors to your home at one time
(vi) Which of the following were listed as symptoms of coronavirus? [Tick all that apply: High temperature; New continuous cough; Blocked or runny nose; Sore throat; Muscle aches; Sneezing; Headache; Pressure in your ears and face; Loss of taste and smell; Shortness of breath]

We counted the number of correct answers participants gave. For questions which had multiple correct answers, a point was awarded if they responded with all the correct answers and no incorrect answers. This gave a comprehension score between 0 and 6.

For most of the questions the answer could be found on the first page of text, sometimes repeated again later, on p.2 of the simplified guidelines or the simplified with visual aids, but later pages [4–7] of the control. For the fifth question, ‘If you are staying at home because you have coronavirus or symptoms of coronavirus, which of these things should you do?’, the four answers were all on p.1–2 of the intervention guidelines but were spread across pages one to six of the control. See Table 1 for details of what page the answers could be found on in each condition.

2. Intention to stay home

**Table 1:** Page of the guidelines on which the answers to each of our primary outcome questions could be found

Question	Answer	Page(s) that the correct answer was on		
		Standard guidelines (Control)	Simplified guidelines	Simplified guidelines with visual aid
What should you do if you have coronavirus or symptoms of coronavirus?	Stay at home and do not leave your house for 7 days after your symptoms started	1	1	1
If you have to stay at home because you have coronavirus or symptoms of coronavirus, how far away should you stay from other people in your home?	2 m or 3 steps	1 & 3	1 & 2	1 & 2
For how many seconds should you wash your hands with soap and water?	20 s	1 & 6	1 & 2	1 & 2
If you have to stay at home because you have coronavirus or symptoms of coronavirus, and then you become more sick and need medical help or advice, what should you do	Contact NHS 111, or 999 in an emergency	1 & 5	1	1
If you are staying at home because you have coronavirus or symptoms of coronavirus, which of these things should you do?	Ask other people to help you get things you need	1 & 2	2	2
	Have food and groceries delivered to you	1 & 2	2	2
	Use disinfectant or household cleaner to regularly clean surfaces	4–5	1 & 2	1 & 2
	Stay away from other people, especially older people	1 & 3	1 & 2	1 & 2
Which of the following were listed as symptoms of coronavirus?	High temperature, new continuous cough	1	1	1

We asked, ‘Would you stay at home for at least 7 days after the start of your symptoms if you had coronavirus or symptoms of coronavirus?’ [Multiple choice from: I definitely would stay home for at least 7 days; I would try to stay home for at least 7 days; I definitely would not stay home for at least 7 days; Not sure.]

We coded this as 1 if they answered that they would ‘definitely’ stay at home for 7 days if symptomatic and 0 otherwise, since the behaviour of interest was staying at home.

### Secondary outcomes (the guidance was displayed again while they were answering)

(i) Looking at the guidance again, would you say it is easy to understand [Answer scale = not at all / a little / somewhat / very /extremely]
(ii) Looking at the guidance again, would you say it makes you feel anxious [Answer scale = not at all / a little / somewhat / very /extremely]

We also asked about, but did not analyse, whether the guidance: is confusing, is reassuring, makes you feel you would know what to do if you had coronavirus, makes you feel the government is taking coronavirus seriously.

### Demographics

Participants were asked about their income, rural/urban location, education, smoking status, parental status, and the number and age of the people in their household. The recruitment companies already had age, gender, and which region of the UK the participant lives in. For full questions see Appendix 4.

### Sample size

Based on the Behavioural Insight Team’s experience of running trials, we recruited 600 participants in each condition. Time constraints precluded a full power analysis in advance of the experiment. However, retrospectively, assuming a total R^2^ of 0.15, we calculated that the study had statistical power of 0.99 to detect an effect size of 0.05 (0.05 R^2^ increase) and 0.95 power to detect a 0.01 (0.01 R^2^ increase) effect. Power calculations were done using g*power 3.1 [12].

### Statistical analysis

Comprehension of guidance and reading time was analysed using linear regression, staying at home with symptoms using logistic regression, and evaluation of the guidance’s simplicity and participants’ anxiety using ordered probit, which estimates the cumulative probability of providing a particular response or a lower one. All analysis was conducted in R studio (version 4.0.0).

## Results

### Participants

**Fig. 2:**
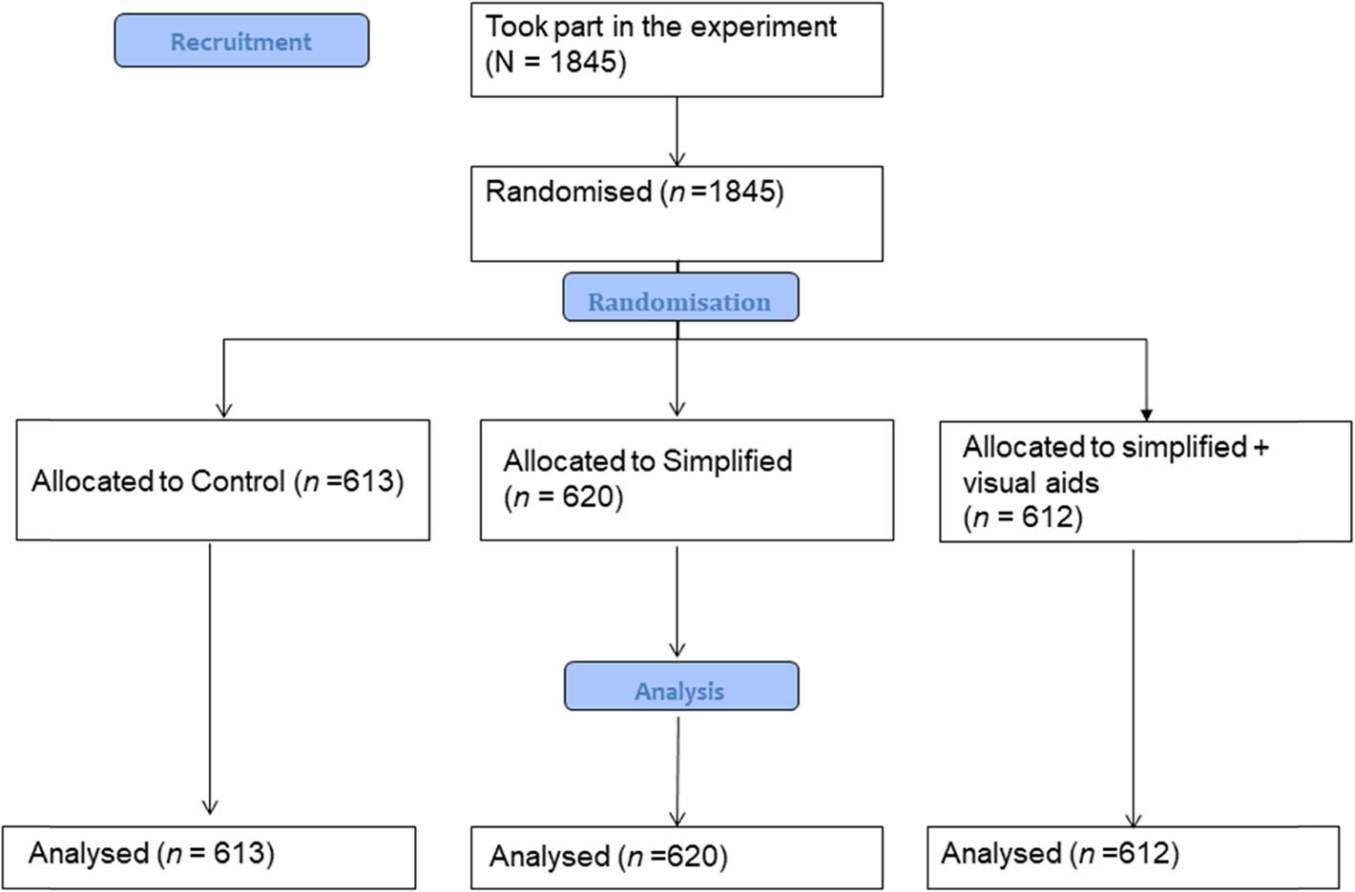
Flowchart showing the randomisation and distribution of participants across conditions

In total, 1845 individuals took part in this experiment. 613 participants were in the control group, 620 in the simplified condition and 612 in the simplified + visual aids condition. Recruitment was from 13 to 16 March 2020, the trial ended when we had reached our pre-specified sample size. No participants were excluded from the analyses. The participant flow is shown in Fig. 2. The mean age was 41, with 909 males (49%), 953 smokers (51%), 822 living with children (44%), 303 who lived alone (16%) and 130 who lived with someone over the age of 69 (7%). There were no significant differences in the distribution of demographics between conditions (see Table 2).

**Table 2:** Variable breakdowns across conditions showing counts and percentages for categorical variables and mean and standard deviation for age; percentages are within treatment

Variables	Control *N* = 613	Simplified *N* = 620	Simplified + visual aids *N* = 612	χ^2^ (df)	*p*
**Continuous**	**Mean / SD**				
Age	40.7 (17.12)	41.41 (17.44)	41.30 (17.04)	–	–
**Categorical**	**N / %**				
Male	323 (53%)	285 (46%)	301 (49%)	5.58 (2)	0.06
Smoker	324 (53%)	297 (48%)	332 (54%)	5.50 (2)	0.06
Living with Children	274 (45%)	267 (43%)	281 (46%)	1.02 (2)	0.60
Living Alone	94 (15%)	112 (18%)	97 (15%)	1.90 (2)	0.39
Living with someone over the age of 69	43 (7%)	47 (7%)	40 (7%)	0.52 (2)	0.77

### Comprehension of the guidelines

The mean comprehension scores did not significantly differ across conditions: 4.27 (1.57) for control, 4.20 (1.55) for simplified and 4.13 (1.55) for simplified + visual aids, F(2, 1842) = 1.235, *p* = 0.291. Similarly, there were no significant differences across condition in the proportions of respondents getting the six individual comprehension questions correct (see Table 3 for full break down of descriptive data for all outcome measures and for the test statistics for the individual questions).

**Table 3:** Breakdown of percentage of correct answers for each question across conditions. Questions 5 and 6 had multiple correct answers, so percentages correspond to those who responded with all and only the correct answers. *P* values correspond to chi-square tests for discrete measures and ANOVA for the continuous measures. No simple comparison was made for the ordinal measures

Question	Overall average (% correct)	Control (% correct)	Simplified (% correct)	Simplified + visual aids (% correct)	*p*
What should you do if you have coronavirus?	69.2%	68%	70%	69%	0.68
How far away should you stay from people?	59.7%	60%	61%	58%	0.46
How long should you wash your hands?	80.8%	83%	81%	79%	0.31
What should you do if you become sicker?	90.1%	91%	90%	89%	0.50
When staying at home, what things should you do?	40.5%	44%	38%	39%	0.054
What are the symptoms of coronavirus?	80.3%	80%	81%	80%	0.86
Average number of correct answers	4.2	4.27	4.20	4.13	0.29
Intention to stay home	84%	85%	83%	84%	0.54
Rating of simplicity (mean / SD)	4.05 (0.95)	4.05 (0.90)	4.05 (1.00)	4.05 (0.95)	–
Rating of anxiety (mean / SD)	2.38 (1.18)	2.37 (1.16)	2.38 (1.2)	2.42 (1.18)	–
Total reading time (median / IQR)	64.97 (140.4)	75.6 (232.6)	64.8 (126.1)	57.6 (112.5)	0.00879

However, when we ran an adjusted regression model to control for demographic variables, there was lower comprehension in the simplified + visual aids condition than in the control, ß = − 0.16, *p* = 0.04998, but the difference between the simplified version and the control remained non-significant, ß = − 0.12, *p* = 0.13. Males (ß = − 0.43, *p* < 0.001), smokers (ß = − 0.36, *p* < 0.001), those living alone (ß = − 0.34, *p* < 0.001) and those with children (ß = − 0.15, *p* = 0.042) all scored significantly lower on comprehension than females, non-smokers, those living with others and those with no children. Older individuals scored higher than younger people (ß = 0.03, *p* < 0.001). These were main effects, which held across conditions. See Table 4 for the full model.

**Table 4:** Results from linear model predicting information recalled from guidance; reference categories are female, non-smoker, living with others, no children, not living with anyone vulnerable and the control condition

Variable	ß	95% CI	T	P
Intercept	3.75	3.50; 4.00	29.32	< 0.001 ***
Simplified	−0.12	−0.29; 0.04	−1.48	0.13
Simplified + visual aids	−0.16	−0.33; − 0.00001	−1.961	0.04998 *
Male	− 0.43	− 0.56; − 0.29	−6.28	< 0.001 ***
Smoker	− 0.36	− 0.50; − 0.22	−5.02	< 0.001 ***
Living alone	− 0.34	− 0.53; − 0.14	−3.305	< 0.001 ***
Has children	− 0.15	− 0.30; − 0.01	−2.04	0.042 *
Living with someone vulnerable	−0.04	− 0.33; − 0.24	−0.31	0.76
Age	0.03	0.02; 0.03	11.83	< 0.001 ***

*p < 0.01

**p < 0.05

***p < 0.001

### Intention to stay home

**Fig. 3:**
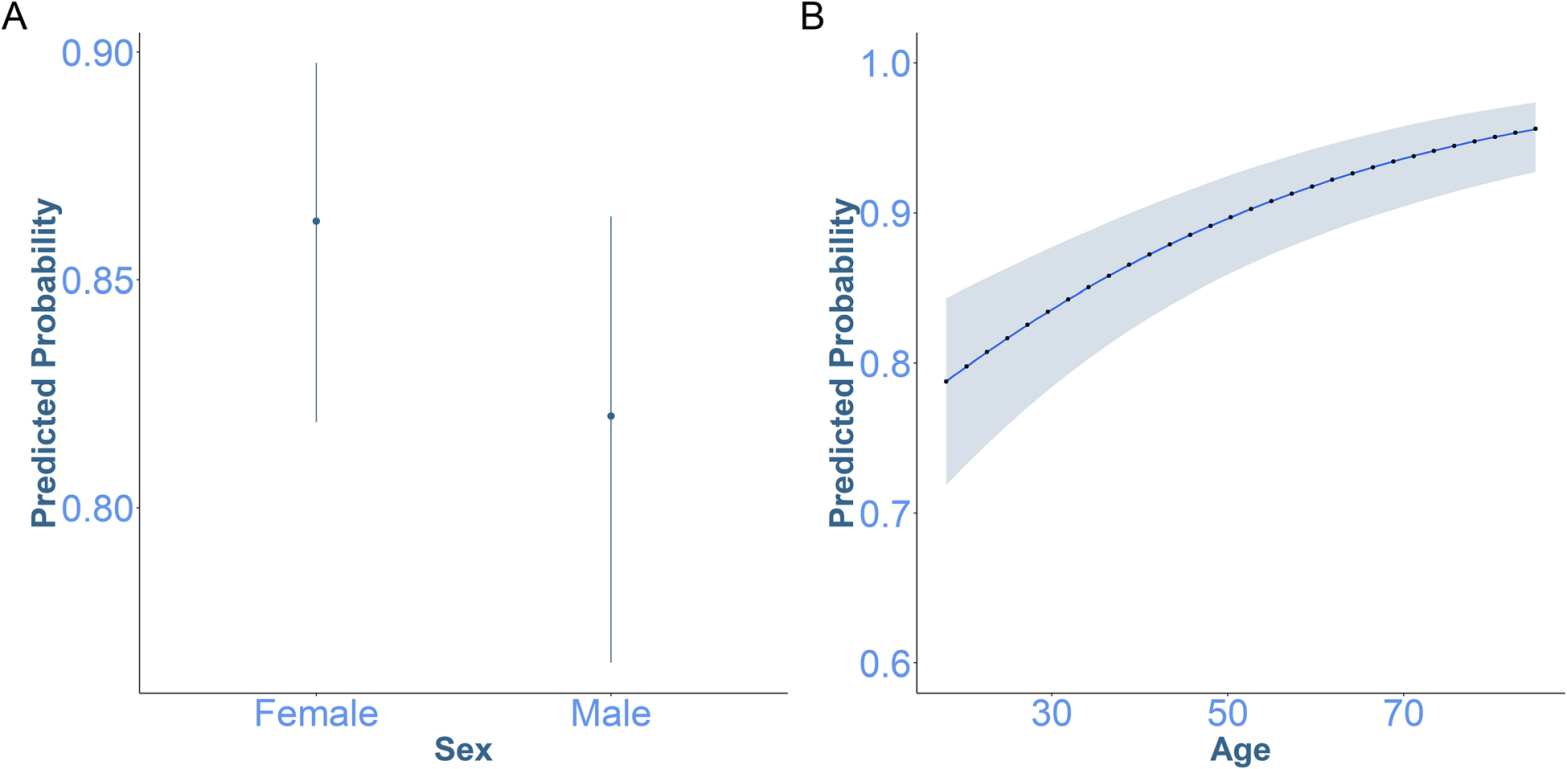
Predicted probabilities of intention to stay home, varying **a** age and **b** sex while holding all other variables constant. Lines and shaded region shows 95% confidence intervals

The percentage of individuals that reported they intended to stay home was 85% in the control, 83% in the simplified and 84% in the simplified + visual aids condition. There were no significant differences in intentions to stay at home between the intervention conditions and the control (simplified: OR = 0.84, 95% CI = 0.61;1.14, *p* = 0.27; simplified + visual aids: OR = 0.89, 95% CI = 0.65, 1.22, *p* = 0.465). This does not change when demographics are included in the model. Males were significantly less likely to intend to stay at home (OR = 0.72, 95% CI = 0.56; 0.94, *p* = 0.015) while older people were more likely (OR = 1.03, 95% CI = 1.02, 1.04, *p* < 0.001). To help visualise the effect sizes, predicted probabilities are shown in Fig. 3. There were no statistically significant effects for the different household types included in the model. See Table 5 for the full model.

**Table 5:** Logistic regression predicting staying at home; reference categories are female, non-smoker, living with others, no children, not living with anyone vulnerable and the control condition

Variable	OR	95% CI	Z	P
Intercept	2.30	1.45; 3.69	3.51	< 0.001 ***
Simplified	0.82	0.60; 1.19	−1.26	0.21
Simplified + visual aids	0.86	0.63; 1.19	−0.90	0.37
Male	0.72	0.56; 0.94	−2.45	0.015 *
Smoker	1.18	0.90; 1.54	1.18	0.24
Living alone	0.79	0.55; 1.15	−1.25	0.22
Has children	1.11	0.84; 1.47	0.76	0.45
Living with someone vulnerable	1.51	0.79; 3.12	1.16	0.25
Age	1.03	1.02; 1.04	5.92	< 0.001

*p < 0.01

**p < 0.05

***p < 0.001

### Ratings of simplicity

The average simplicity rating given by participants was 4.05 (0.90) for control, 4.05 (1.00) for simplified and 4.05 (0.95) for simplified + visual aids.

**Fig. 4:**
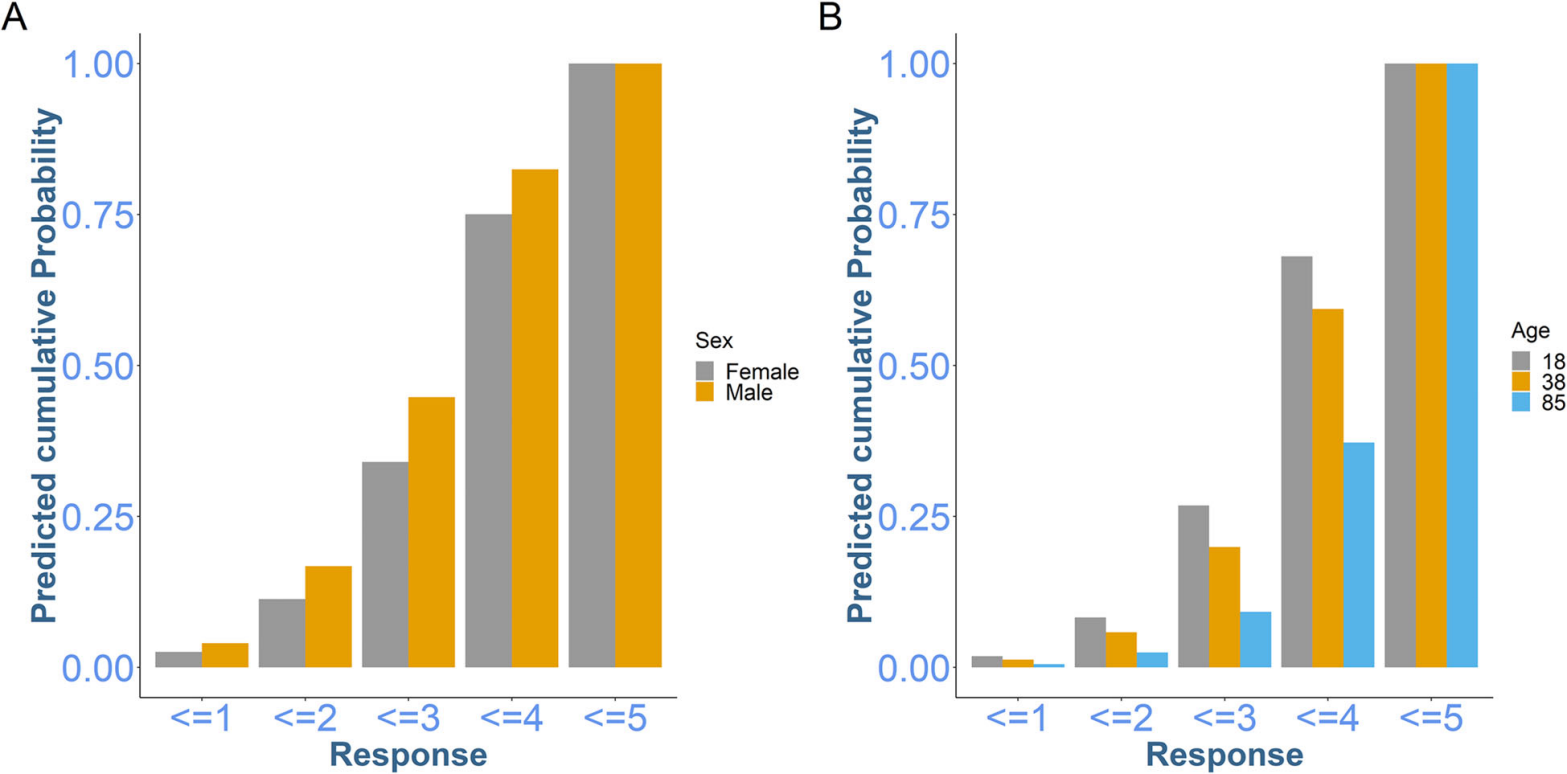
Predicted cumulative probability for simplicity ratings, varying **a** sex and **b** age holding all other variables constant. The values chosen for age are the minimum (18) the median (35) and the maximum (85). Taller bars at lower values indicate greater probability of giving lower responses

There was no significant difference in ratings of simplicity between the control and simplified guidance (OR = 1.05, 95% CI = 0.86; 1.30, *p* = 0.63) or between the control and simplified + visual aids (OR = 1.01, 95% CI = 0.82; 1.25, *p* = 0.91). Older people rated the guidance as easier to understand (OR = 1.02, 95% CI = 1.014; 1.025, *p* < 0.001) while males rated it as more difficult to understand (OR = 0.64, 95% CI = 0.54; 0.76, *p* < 0.001). Predicted cumulative probabilities for age and sex are given in Fig. 4. None of the other demographic variables were statistically significant. For the full model, see Table 6.

**Table 6:** Results from ordered probit model predicting ratings of ease of understanding (1 = not at all easy, 5 = extremely easy); cut points show the intercepts for a score that was of equal to or greater than the value. 1 is absent as this is, by definition, infinite

Cut points	OR	95% CI	P
= > 2	37.58	23.47; 60.19	< 0.001 ***
= > 3	7.81	5.50; 11.10	< 0.001 ***
= > 4	1.94	1.40; 2.68	< 0.001 ***
= > 5	0.33	1.40; 0.46	< 0.001 ***
**Variables**			
Simplified	1.05	0.86; 1.30	0.63
Simplified + visual aids	1.01	0.82; 1.25	0.91
Male	0.64	0.54; 0.76	< 0.001 ***
Smoker	1.07	0.89; 1.28	0.50
Living alone	0.94	0.73; 1.21	0.64
Has children	0.89	0.74; 1.08	0.24
Living with someone vulnerable	0.85	0.59; 1.21	0.36
Age	1.02	1.014; 1.025	< 0.001 ***

*p < 0.01

**p < 0.05

***p < 0.001

### Ratings of anxiety

**Fig. 5:**
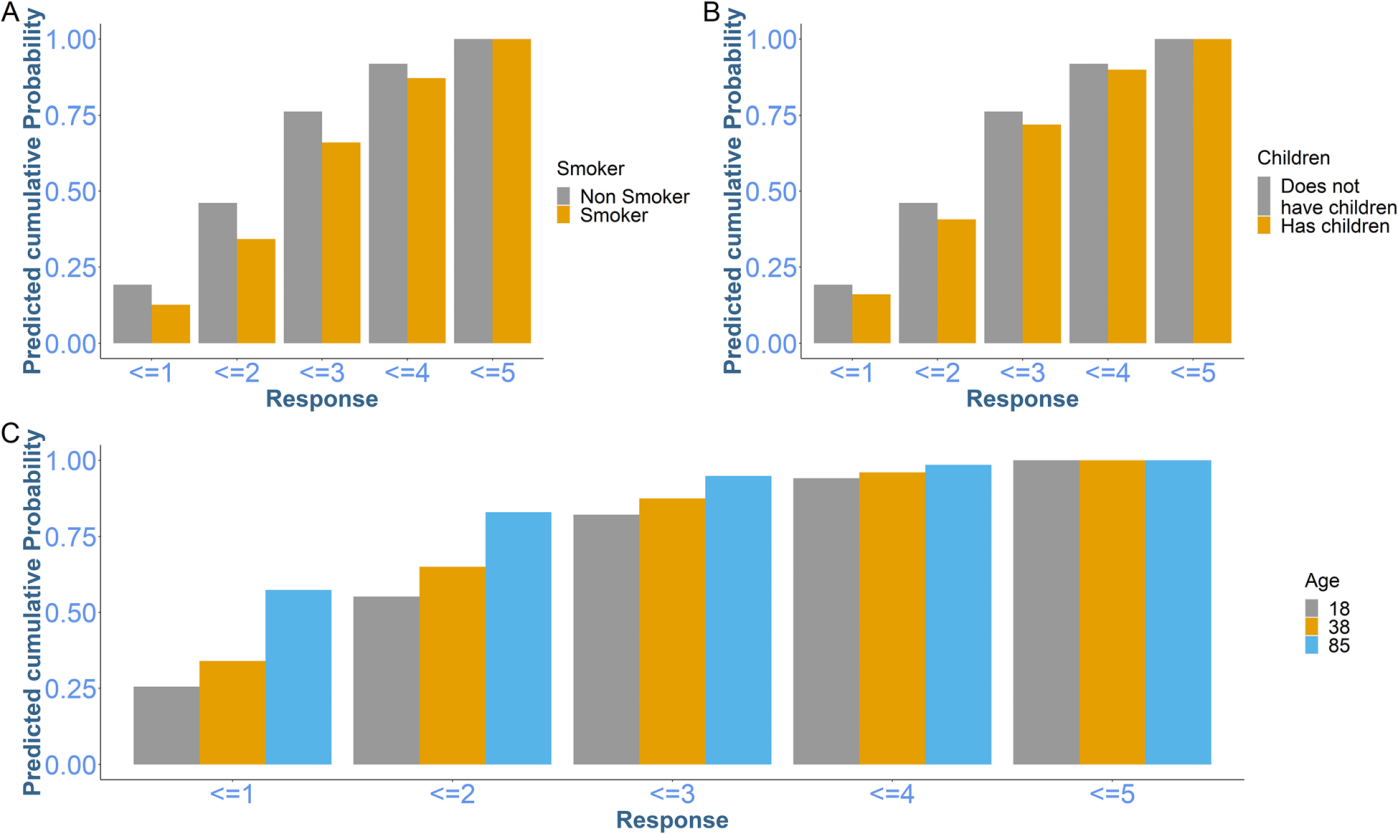
Predicted cumulative probability of anxiety, varying **a** parental status, **b** smoking and **c** age holding all other variables constant. The values chosen for age are the minimum (18) the median (35) and the maximum (85). Taller bars at lower values indicate greater probability of giving lower responses

The mean and standard deviation in ratings of how anxious the guidance made participants feel was 2.37 (1.16) for control, 2.38 (1.2) for simplified and 2.42 (1.18) for simplified + visual aids. There was no significant difference between either simplified (OR = 1.04, 95% CI = 0.85; 1.23, *p* = 0.71) or simplified + visual aids (OR = 1.11, 95% CI = 0.91; 1.36) and the control. Older people reported significantly less anxiety (OR = 0.98, 95% CI = 0.97; 0.99, *p* < 0.001) while smokers (OR = 1.64, 95% CI = 1.38; 1.96, *p* < 0.001) and those with children (OR = 1.24, 95% CI = 1.04; 1.49, *p* = 0.02) reported significantly more. Cumulative probabilities are displayed for age, smoking status, and having children in Fig. 5. The full model is shown in Table 7.

**Table 7:** Results from ordered probit model predicting ratings of anxiety from the guidance (1 = not at all, 5 = extremely high). Cut points show the intercepts for a score that was equal to or greater than the value

Cut points	OR	95% CI	P
= > 2	4.22	3.05; 5.83	< 0.001 ***
= > 3	1.17	0.85; 1.60	0.33
= > 4	0.31	0.23; 0.43	< 0.001 ***
= > 5	0.09	0.06; 0.13	< 0.001 ***
**Variables**			
Simplified	1.04	0.85; 1.23	0.71
Simplified + visual aids	1.11	0.91; 1.36	0.32
Male	0.99	0.84; 1.17	0.92
Smoker	1.64	1.38; 1.96	< 0.001 ***
Living alone	0.89	0.70; 1.14	0.34
Has children	1.24	1.04; 1.49	0.02 *
Living with someone vulnerable	1.25	0.88; 1.76	0.21
Age	0.98	0.97; 0.99	< 0.001 ***

*p < 0.01

**p < 0.05

***p < 0.001

### Reading time

There were differences in reading time across conditions. The median and interquartile ranges for each condition were: control = 75.6 s (232.6); simplified = 64.8 s (126.1); simplified + visual aids = 57.6 s (112.5), F(2, 1842) = 4.746, *p* = 0.00879. (Given substantial skew on this variable, log transformations were conducted for all analyses.) A Tukey post hoc test revealed participants read for longer in the control condition compared to the simplified + visual aids condition (diff = − 0.20, *p* = 0.0101), but there was no significant difference between control and simplified (*p* = 0.0523) or simplified and simplified + visual aids (*p* = 0.822). When demographic variables were added, both the simplified (ß = − 0.21, *p* = 0.0011) and simplified + visual aids (ß = − 0.22, *p* < 0.001) conditions were associated with significantly less reading time than the control. Males (ß = − 0.31, *p* < 0.001) and smokers (ß = − 0.21, *p* < 0.001) were spent less time reading the guidance, while older individuals spent longer (ß = 0.025, *p* < 0.001). See Table 8 for the full model.

**Table 8:** Linear model predicting log reading time. Reference categories are female, non-smoker, living with others, no children, not living with anyone vulnerable and the control condition

Variable	ß	95% CI	T	P
Intercept	3.57	3.38; 3.77	36.049	< 0.001 ***
Simplified	−0.21	−0.34; −0.08	−3.268	0.0011 **
Simplified + visual aids	− 0.22	− 0.35; − 0.10	−3.531	< 0.001 ***
Male	− 0.31	0.42; − 0.21	−5.96	< 0.001 ***
Smoker	− 0.21	− 0.32; − 0.10	−3.741	< 0.001 ***
Living alone	− 0.058	− 0.21; 0.09	−0.74	0.46
Has children	0.022	−0.09; 0.13	0.376	0.71
Living with someone vulnerable	0.038	−0.18; − 0.10	0.342	0.73
Age	0.025	0.021; 0.028	14.393	< 0.001 ***

*p < 0.01

**p < 0.05

***p < 0.001

### Reading time and comprehension

Reading time predicted comprehension of the guidance in linear regression, (ß = 0.65, *p* < 0.001). Among the demographic variables, both males and smokers tended to have shorter reading times and to have a lower comprehension of the guidance, while being older predicted both a longer reader time and greater comprehension. Mediation analysis [13] shows reading time partially (but not fully) mediates the effect of the demographic variables on comprehension, as can be seen from the models in Table 9. Compared to Model 1, when reading time was added into the model (Model 2) the coefficients were reduced, but remained statistically significant, suggesting some, but not all, of the variance in comprehension for these demographics is being explained by reading time.

**Table 9:** Linear regression predicting comprehension from males, smoking and age with and without (log) reading time in seconds

	Model 1: Does not include reading time		Model 2: Includes total reading time	
Variable	ß (95% CI)	*p*	ß (95% CI)	*p*
Simplified	−0.13 (− 0.29; 0.03)	0.12	0.009 (− 0.13; 0.15)	0.9
Simplified + visual aids	−0.17 (− 0.33; − 0.003)	0.047 *	−0.01 (− 0.16; 0.12)	0.8
Male	−0.43 (− 0.57; − 0.30)	< 0.001 ***	−0.23 (− 0.35; − 0.11)	< 0.001 ***
Smoker	−0.38 (− 0.52; − 0.24)	< 0.001 ***	−0.25 (− 0.37; − 0.13)	< 0.001 ***
Age	0.025 (0.021; 0.030)	< 0.001 ***	0.009 (0.005; 0.01)	< 0.001 ***
Total reading time	–	–	0.66 (0.61; 0.71)	< 0.001 ***

*p < 0.01

**p < 0.05

***p < 0.001

When total reading time was added into the model (Model 2) the coefficient for the Simplified + visual aids decreased and was no longer significant (Model 1: ß = − 0.17, *p* = 0.047; Model 2: ß = − 0.01, *p* = 0.8). This suggests that total reading time fully mediated the difference in comprehension [13].

## Discussion

There were no differences in comprehension between the three different versions of the guidelines in the unadjusted models, with the Control group averaging 4.27, the Simplified 4.20, and the Simplified + visual aids 4.13 out of a possible total of 6 correct answers. However, when the model was adjusted for demographic variables, there was lower comprehension in the simplified + visual aids condition than in the control, (ß = − 0.16, *p* = 0.04998). Males, smokers, those living alone, and those with children scored less on comprehension than females, non-smokers, those living with others and those with no children. Older individuals scored higher than younger people.

Participants spent longer reading the control guidance than the two simplified versions (75.6 s vs 64.8 s and 57.6 s). However, the control guidance was approximately two and a half times longer than the interventions and participants only spent 30% longer reading it. We cannot be certain that participants read until the end. The answers to most of the questions could be found on the first two pages. However, the control group did as well as the intervention groups on the question about what to do if you have to stay home, some of whose answers were to be found on pages 4–7 of the guidance. If participants read all the way through the guidance in each condition, then their reading speed would have been faster in the control than the simplified versions. It may be more likely that participants skim read and scanned for important information.

Males and smokers spent less time reading, and older participants spent more time. When reading time, comprehension, and demographics were entered into the same model, total reading time fully mediated the negative relationship between the simplified + visual aids guidance and comprehension, suggesting that the decrease in comprehension of that guidance was caused by the participants’ shorter reading time. Total reading time partially mediated the relationship between comprehension and sex, smoking status, and age, suggesting that differences in comprehension among these groups is partly explained by differences in reading time.

Our finding that reading time drives differences in comprehension but text simplification has either no effect or a negative effect is surprising, especially considering that there is a body of evidence that comprehension can be improved by making changes to increase the text cohesion, e.g., adding headers and topic sentences to mark out key concepts [8, 9]. However, our findings are consistent with research showing that text simplification may benefit those with low background knowledge while having a negative effect on the comprehension of those with high background knowledge [8]; the apparent ease of readability of texts may lead readers to process them less deeply, with the simplicity being a signal that less effort is required [8]. In the case of our simplified guidance with visual aids, which had a negative effect, it may be that participants were looking at the pictures instead of reading the text.

Alternatively, despite being longer, the control guidelines might already have been simple in the ways that matter for reading comprehension. Considerable effort is made in this regard, including the involvement of internal Communications and Behavioural Science teams, to ensure that rapidly published public health advice is succinct, clear and actionable. The simplified versions were not rated as any simpler than the control by our participants. Psychological research shows that the more precisely behaviours are specified, the more they are likely to be carried out and that rewriting guidelines with specific instructions in plain English may be the simplest, most effective method of increasing implementation [14, 15]. The Government Digital Service (GDS) Style Guide already incorporates this advice [16]. The control version would already have been based on GDS style, so it may already have incorporated the most important principles for reading comprehension.

Varying the way that the guidance was presented did not affect participants’ intention to stay home if they had symptoms, which averaged 84% over the sample. Nor did it change participants’ perception of their ease of understanding (which was 4.05 in each condition) or the anxiety they felt on reading the guidance, which was a little/ somewhat (averaged 2.38). This result is consistent with a French trial, which found that simplifying two posters designed to promote preventive behaviours and handwashing, by streamlining them, had no effect on intention to perform preventative behaviours [17]. Nevertheless, we do know that receiving communications is important: a Chinese national cross-sectional survey found that exposure to risk communication messages was positively associated with engaging in preventive behaviours [18].

Although we found stated intention to stay home was high, it is well known that intentions are not necessarily a good predictor of behaviour [19]. There is evidence of this specifically with regard to self-isolation. A longitudinal survey in the UK found that, of participants who had not had covid symptoms in the past 7 days, the intention not to leave home if they developed symptoms was around 70% from 2nd March – 5th August 2020; however, of those who reported having had covid symptoms in the past 7 days, only 18.2% said they had not left home since developing the symptoms [20]. The same survey found a similar demographic pattern to intentions as we did: males and younger people were less likely to report adhering to guidelines.

Although participants on average rated the guidance as ‘very’ easy to understand, they only answered two thirds of our comprehension questions correctly on average. This could be due to the relatively short time participants spent reading the guidance (participants on average spent roughly a minute reading the three-side versions and 75 s on the seven-side version); however, it is also well established in the literature on text comprehension that people’s subjective self-rated comprehension does not correlate well with their actual performance on objective comprehension measures [21–23].

The strength of this study is its large sample size, which meant we were highly powered to find even a very small result. The main limitation is the potential lack of external validity of the participant pool. Our participants were panel members engaging in our task for money. Since they were drawn from a self-selecting group who had agreed to be on a panel and answer surveys for money, their behaviour may not be representative of the average member of the population. One reason we paid them a bonus for each correct answer is that our experience suggested that payment would be necessary to induce them to pay attention to the study materials (even though the pandemic was a national crisis that the participants were in). Arguably, that means our results underestimate comprehension of the guidance by its real-life intended readers, since those people would have sought out the guidance on gov.uk and therefore presumably have been motivated to read it. We would expect the difference in motivation to affect comprehension, since the activation of knowledge in memory and the integration of text-based information with that knowledge are guided by the reader’s goals and pre-existing sense of what constitutes adequate comprehension [24, 25].

## Conclusion

Simplified guidance did not improve comprehension compared to the standard long-form guidance we used as a control, and simplified guidance with visual aids may even have worsened comprehension. The simplified guidance did not affect people’s intention to comply with the guidance and stay home if they had covid symptoms. Further research is required on the effect of simplified texts in emergency guidance. Simplicity may not always be a good thing in a complex and changing situation.

## Supplementary Information

**Additional file 1.**

**Additional file 2.**

**Additional file 3.**

**Additional file 4.**

## Data Availability

The datasets used and/or analysed during the current study are available from the corresponding author on reasonable request.

## Abbreviations

CI: Confidence interval
FAQ: Frequently Asked Question
GDS: Government Digital Service
OR: Odds ratio
PHE: Public Health England
UK: United Kingdom

## Footnotes

1 Predictiv is an end-to-end platform that aims to make online experiments accessible to policy makers and other organisations driven by social impact. The platform provides functionality to run economic experiments and has access to a large international panel, including 200,000 people in the UK and 1 million in the US, through a network of online panel suppliers. More information can be found on www.predictiv.co.uk.
