# Supplementary material for: A randomized controlled trial to test the effect of simplified guidance with visuals on comprehension of COVID-19 guidelines and intention to stay home if symptomatic
Source: BMC Public Health. 2021 May 10;21:892. doi: 10.1186/s12889-021-10787-9 (PMC8108739; doi:10.1186/s12889-021-10787-9)
Supplement: Supplementary file 1 — Additional file 1. [file 12889_2021_10787_MOESM1_ESM.docx]

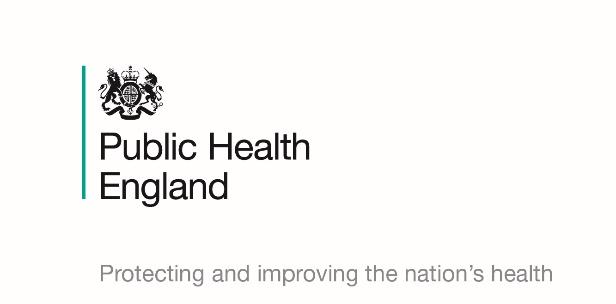


Stay at Home:

Guidance for people with confirmed or possible coronavirus infection (COVID-19)

**Document Owner:** Guidance Cell

| **Organisations consulted** | **Date sent for comments** | **Version number** | **Date feedback received** |
| --- | --- | --- | --- |
| CMO, PHE, NHSE | 10 March | V 0.01 | 10 March |
| PHE | 11/03/2020 | V0.02 |  |
|  |  |  |  |
|  |  |  |  |
|  |  |  |  |
|  |  |  |  |

**Accredited and endorsement:** Pending

**Publication date**: Pending

**Symptoms**

The most common symptoms of coronavirus (COVID-19) are recent onset of:

- New continuous cough
- High temperature^^[[1]](#footnote-1)^^

For most people, coronavirus (COVID-19) will be a mild infection

**Key messages**

- If you have symptoms of coronavirus infection (COVID-19), stay at home and do not leave your house for 7 days from when your symptoms started. (See Ending Isolation section below for more information)
- This action will help protect others in your community whilst you are infectious.
- Plan ahead and ask others for help to ensure that you can successfully stay at home.
- Ask your employer, friends and family to help you to get the things you need to stay at home.
- Stay at least 2 metres (about 3 steps) away from other people in your home whenever possible.
- Sleep alone, if that is possible.
- Wash your hands regularly for 20 seconds, each time using soap and water.
- Stay away from vulnerable individuals such as the elderly and those with underlying health conditions as much as possible.
- If your symptoms worsen during home isolation or are no better after 7 days use NHS 111 online or call NHS 111; for a medical emergency dial 999.

**Who this guidance is for**

This advice is intended for people with symptoms of coronavirus (COVID-19), including those with a diagnosis of coronavirus (COVID-19) infection, who must remain at home until they are well.

**Will I be tested if I think I have COVID-19**

We will not be testing those self-isolating with mild symptoms. The following advice is designed to help people prevent the spread.

**Why has the advice on self-isolation changed from 14 days to 7 days**

The 14 day period is for those who have had exposure to a confirmed case but have not shown symptoms. The 14 days represents the potential incubation period (the time it takes for symptoms to show if you have been infected).

Most people will no longer be likely to transmit the virus 7 days after the onset of symptoms. If your symptoms persist past 7 days you should contact NHS 111 online at 111.nhs.uk. If you have no internet access, you should call NHS 111.

**Why staying at home is very important**

It is very important that you stay at home whilst you have coronavirus (COVID-19). This will help to protect your friends, colleagues and the wider community and will help control the spread of the virus.

We realise that staying at home may be difficult or frustrating, but there are things that you can do to help make it easier. These include:

- Plan ahead and think about what you will need in order to be able to stay at home for the full 7 days.
- Talk to your employer, friends and family to ask for their help to access the things you will need in order to successfully stay at home.
- Think about and plan how you can get access to food and other supplies such as medications that you will need during this period.
- Ask friends or family to drop off anything you need or order supplies online, but make sure these are left outside your home for you to collect.
- Make sure that you keep in touch with friends and family over the phone or through social media.
- Think about things you can do during your time at home. People who have successfully completed a period of staying at home have kept themselves busy with activities such as cooking, reading, online learning and watching films.
- When you are feeling better, remember that physical exercise can be good for your wellbeing. Look for online classes or courses that can help you take light exercise in your home.

**While you are staying at home, make sure you do the following things:**

**Stay at home**

You should remain in your home. Do not go to work, school, or public areas, and do not use public transport or taxis. You cannot go for a walk.

You will need to ask friends or relatives if you require help with buying groceries, other shopping or picking up medication. Alternatively, you can order medication by phone or online. You can also order your shopping online. Make sure you tell delivery drivers to leave items outside for collection if you order online. The delivery driver should not come into your home.

**At home, try as best you can to separate yourself from the people you live with**

You should aim to stay in a well-ventilated room with a window that can be opened. Try to keep the window open as much as possible to enable ventilation and air flow as this will help to keep clean air moving through your room.

Try to separate yourself from other people in your home and keep the door closed. If you cannot stay in a separate room aim to keep 2 metres (3 steps) away from the other people in your house. We understand that this can be particularly difficult for parents of small children, people with other caring responsibilities or those living in studio apartments, for example. Further advice can be found below. Try to keep yourself separated from other people as much as possible, and make sure everyone regularly follows the steps in this document (washing your hands, not touching your face, cleaning surfaces etc.).

Use your own toothbrushes, eating and drinking utensils (including cups and glasses in the bathroom and bedroom), dishes, drinks, towels, washcloths or bedlinen.

If you have your own garden it is fine to use it as long as you keep two metres away from other members of your household. If possible they should use the outside area separately.

**Use of shared spaces if you live with others**

Minimise the time you spend in shared spaces such as kitchens, bathrooms and sitting areas as much as possible and keep shared spaces well ventilated.

Aim to keep 2 metres (3 steps) away from people you live with and sleep in a different bed where possible. If you can use a separate bathroom from the rest of the household. Make sure you use separate towels from other people in your house, both for drying yourself after bathing or showering and for hand hygiene purposes. Ask your family or the people you live with to remember to use their own towels.

If you do share toilet and bathroom, it is important that you clean them after you have used them every time (e.g. wiping surfaces you have come in contact with). Another tip is to consider drawing up a rota for bathing, with you using the facilities last, before thoroughly cleaning the bath, sink and toilet yourself.

If you share a kitchen with others, avoid using it whilst others are present. Take your meals back to your room to eat. If you have one, use a dishwasher to clean and dry your used crockery and cutlery. If this is not possible, wash them using your usual washing up liquid and warm water and dry them thoroughly, remembering to use a separate tea towel.

We understand that it will be difficult for some people to separate themselves from others at home. You should do your very best to follow this guidance and everyone in your household should regularly wash their hands, avoid touching their face and clean frequently touched surfaces.

**What if I am living alone with my children?**

Keep following this advice to the best of your ability however we are aware that not all these measures will be possible.

What we have seen so far is that children with COVID-19 appear to be less severely affected. It is nevertheless important to do your best to follow this guidance.

If your child develops symptoms, they need to stay at home for 7 days from the onset of their symptoms.

**What if I live with an elderly, vulnerable or pregnant person?**

If you provide care to an elderly, vulnerable or pregnant person keep following this advice to the best of your ability.

However, we are aware that not all these measures will be possible.

**What if I am breastfeeding while infected?**

There is currently no clinical evidence to suggest that the virus can be transmitted through breast milk. Infection can be spread to the baby in the same way as to anyone in close contact with you. We believe that the benefits of breastfeeding outweigh any potential risks of transmission of the virus through breastmilk or by being in close contact, however this will be an individual decision and can be discussed with your midwife or doctor by telephone.

If you wish to breastfeed, you should take precautions to limit potential spread of COVID-19 to the baby:

- Wash your hands before touching the baby, breast pump or bottles;
- Wear a face-mask for feeding at the breast;
- If you use a breast pump, clean it as recommended by the manufacturer after each use;
- Consider asking someone who is well to feed your expressed breast milk to the baby

If you are feeding with formula or expressed milk, you should sterilise the equipment carefully before each use. You should not share bottles or a breast pump with someone else.

You can find more information [Royal College of Obstetricians and Gynaecologists website](https://www.rcog.org.uk/en/guidelines-research-services/guidelines/coronavirus-pregnancy/covid-19-virus-infection-and-pregnancy/).

**Cleaning and disposal of waste**

When cleaning you should use your usual household products, like detergents and bleach as these will be very effective at getting rid of the virus on surfaces. Clean frequently touched surfaces.

Personal waste (e.g. used tissues) and disposable cleaning cloths can be stored securely within disposable rubbish bags. These bags should be placed into another bag, tied securely and kept separate from other waste within your own room. This should be put aside for at least 72 hours before being put in your usual external household waste bin.

Other household waste can be disposed of as normal.

**Laundry**

Do not shake dirty laundry; this minimises the possibility of dispersing virus through the air.

Wash items as appropriate in accordance with the manufacturer’s instructions.. Dirty laundry that has been in contact with an ill person can be washed with other people’s items.

If you do not have a washing machine you can then take your laundry to a launderette after your isolation period has ended.

If you do not have a washing machine wait a further 72 hours after your 7 day isolation period has ended when you can then take your laundry to a public laundromat.

**What you can do to help yourself get better**

Drink water to keep yourself hydrated; you should drink enough during the day so your urine (pee) is a pale clear colour. You can use over the counter medications, such as paracetamol, to help with some of your symptoms. Use these according to the instructions on the packet/label and do not exceed the recommended dose.

**If you need to seek medical advice**

Seek prompt medical attention if your illness is worsening. If it’s not an emergency, you should us NHS 11 online or call NHS 111. If it is an emergency and you need to call an ambulance, dial 999 and inform the call handler or operator that you have coronavirus (COVID-19).

All routine medical and dental appointments should usually be cancelled whilst you are sick and staying at home. If you are concerned or have been asked to attend in person within the period you are home isolating, discuss this with your medical contact first (e.g. your GP, local hospital or outpatient service), using the number they have provided. If your concerns are related to COVID-19 contact NHS 111 online at 111.nhs.uk. If you have no internet access, you should call NHS 111.

.

**Wash your hands often**

Cleaning your hands frequently throughout the day by washing with soap and water for 20 seconds or using hand sanitiser will help protect you and the people you live with. This step is one of the most effective ways of reducing the risk of infection to you and to other people.

**Cover your coughs and sneezes**

Cover your mouth and nose with disposable tissues when you cough or sneeze.

If you have a carer they should take care to use disposable tissues to wipe away any mucus or phlegm after you have sneezed or coughed.

Dispose of tissues into a disposable rubbish bag and immediately wash your hands with soap and water or use a hand sanitiser.

**Facemasks**

We do not recommend the use of facemasks as an effective means of preventing the spread of infection. Facemasks play an important role in clinical settings, such as hospitals, but there’s very little evidence of benefit from their use outside of these settings. However if you receive external care you may be asked to wear a mask to minimise the risk to your carer.

**Do not have visitors in your home**

Do not invite or allow social visitors, such as friends and family, to enter your home. If you want to speak to someone who is not a member of your household, use the phone or social media.

**If you have pets in the household**

. At present, there is no evidence that companion animals/pets such as dogs and cats can be infected with coronavirus (COVID-19).

**Looking after your wellbeing whilst staying at home**

We know that staying at home for a prolonged period of time can be difficult, frustrating and lonely for some people and that you may feel low. It's important to remember to take care of your mind as well as your body and to get support if and when you need it. Stay in touch with family and friends over the phone or on social media. There are also sources of support and information that can help

<https://www.nhs.uk/oneyou/every-mind-matters/>

Think about things you can do during your time at home. People who have successfully completed a period of staying at home have kept themselves busy with activities such as cooking, reading, online learning and watching films. If you feel well enough you can take part in light exercise within your home or garden.

**Ending self-isolation**

You should remain at home until 7 days after the onset of your symptoms. After 7 days, if you feel better and no longer have a fever, you can return to your normal routine. If you have not had any signs of improvement and have not already sought medical advice, you should contact NHS 111 online or by phone before you leave your home or let visitors in.

Cough may persist for several weeks in some people, despite the coronavirus infection having cleared. A persistent cough alone does not mean you must continue to self-isolate for more than 7 days.

1. Temperature of 37.8 degrees centigrade or higher [↑](#footnote-ref-1)
