# Supplementary material for: A randomized controlled trial to test the effect of simplified guidance with visuals on comprehension of COVID-19 guidelines and intention to stay home if symptomatic
Source: BMC Public Health. 2021 May 10;21:892. doi: 10.1186/s12889-021-10787-9 (PMC8108739; doi:10.1186/s12889-021-10787-9)
Supplement: Supplementary file 2 — Additional file 2. [file 12889_2021_10787_MOESM2_ESM.docx]

# Stay at home: Guidance for people with confirmed or possible coronavirus

**If you have coronavirus (COVID-19), or any symptoms of coronavirus, you must stay at home for 7 days after your symptoms start**. You need to do this even if you have mild symptoms. The two main coronavirus symptoms are:

1. A new, continuous cough

2. High temperature (37.8C or higher)

Most people with coronavirus or symptoms of coronavirus will recover safely at home. However, **if you are staying at home and your symptoms are getting worse, or if you are ill for more than 7 days, go to 111.nhs.uk or call 111.** If it is an emergency, call 999 and say you have coronavirus.

## **Staying at home: What you need to do (summary)**

**

**

## **Staying at home: What you need to do (details)**

If you have coronavirus, or any symptoms of coronavirus, you must stay at home for 7 days after your symptoms start. There are 5 main things you should do during this time:

**1. Stay at home**

- Do not go to work, school or public areas
- Do not use public transport or taxis
- Do not have visitors to your home
- Cancel your routine medical/dentist appointments
- Ask friends or relatives to help you get groceries or medication, or buy these online. Have deliveries left outside your door.

**2. Practice good hygiene**

- Wash your hands regularly for 20 seconds. Use soap and water or hand sanitiser.
- When you cough or sneeze, use a tissue to cover your mouth and nose. Put the tissue in the bin immediately, and wash your hands.
- Ask the people you live with to do all these things also

**3. Stay away from other people**

- Stay at least 2 metres (about 3 steps) away from other people.
- If possible, stay in a different room with the door closed. Open the window regularly to ventilate the room.
- If possible, sleep and eat alone in a separate room
- If possible, use a separate bathroom. If you can’t, clean the shared toilets and bathrooms each time after you use it.
- Use your own toothbrush, utensils, dishes, towels, washcloths and bedlinen.
- You can go into your garden, but stay at least 2 metres away from others.

**4. Clean and dispose of waste**

- Clean surfaces often using detergents and bleach
- Put used tissues and disposable cleaning cloths in rubbish bags. Then, put these bags into another bag, tie securely and keep separate from other waste. Put this bag aside for 72 hours before putting it in your usual household waste bin
- Wash your own dishes, bedding, towels and clothing separately. Do not shake your dirty laundry - this can spread the virus.

**5. Take care of yourself**

- Drink plenty of fluids - your urine should be a pale clear colour.
- Use over the counter medicines like paracetamol for any pain.
- Stay busy. Use your phone or social media to keep in touch with friends and family. Keep doing things you like, such as cooking, reading, online learning and watching films.
- If you can, do some light exercise in your home when you are feeling better
- If you feel low, get support at [Every Mind Matters](https://www.nhs.uk/oneyou/every-mind-matters/)

## **Frequently Asked Questions**

| **Common questions** | **Answer** |
| --- | --- |
| *Will I be tested if I think I have coronavirus?* | There are currently no plans to test people who are staying at home because of coronavirus. |
| *Why has the advice on self-isolation changed from 14 days to 7 days?* | You need to stay at home for 7 days if you   - have coronavirus, or - have either of the two main symptoms of coronavirus, even if they are mild   You need to stay at home for 14 days if   - you do not currently have coronavirus or any symptoms of coronavirus, but you have recently been exposed to a person with confirmed coronavirus. |
| *What if I am living alone with my children?* | Children appear to be less severely affected than adults by coronavirus. However, please follow this advice as much as you can. We understand this will be more difficult with a child. If your child develops symptoms, they will also need to stay at home for 7 days after their symptoms start. |
| *What if I live with an elderly, vulnerable or pregnant person?* | Follow this advice as much as you can. However, we understand not all these measures may be possible to do in this situation. |
| *What if I am breastfeeding while infected?* | There is currently no clinical evidence to suggest that the virus can be transmitted through breast milk. However, babies can get the virus in the same way as other people. So, take these precautions when breastfeeding:   - Wash your hands before touching the baby, breast pump or bottles; - Wear a facemask for feeding at the breast; - Clean your breast pump as recommended by the manufacturer after each use; - Ask someone who is not ill to feed your expressed breast milk to the baby   If you are feeding with formula or expressed milk, sterilise the equipment before each use. Do not share bottles or a breast pump with someone else. |
