# Supplementary material for: A randomized controlled trial to test the effect of simplified guidance with visuals on comprehension of COVID-19 guidelines and intention to stay home if symptomatic
Source: BMC Public Health. 2021 May 10;21:892. doi: 10.1186/s12889-021-10787-9 (PMC8108739; doi:10.1186/s12889-021-10787-9)
Supplement: Supplementary file 4 — Additional file 4. [file 12889_2021_10787_MOESM4_ESM.pdf]

Testing the efficacy of coronavirus messaging

Experiment 1: Guidance for people with confirmed or possible coronavirus infection
13 March 2020

THE BEHAVIOURAL INSIGHTS TEAM

In partnership with

Cabinet Office

PREDICTIV

Experiment page 1
Introduction I

Welcome and thank you for participating in this survey.

Task: In this exercise, we are going to show you some information about the coronavirus. You can look at this information for as long as you like. We are then going to ask you some questions about the information you saw.

Duration: The survey should take about 12 minutes to complete and requires your attention, so please only participate if you can dedicate this time!

Experiment page 1
Introduction II

Payment: For 6 questions in this survey, **you can earn an additional reward of £0.30 for each correct answer, up to a maximum of £1.80**. This reward is in addition to the reward you get from completing the survey. You will receive this after completing the survey.

You are about to participate in a survey being run by The Behavioural Insights Team for Public Health England. Any responses you submit in this survey are anonymous. This means we do not collect personally identifiable information from you, and we will not be able to connect any personally identifiable information to your answers in this survey. As part of the study, we may share an anonymised dataset with Public Health England. In addition, we may retain your responses and use them in other projects that promote scientific knowledge (such as an academic publication). You can withdraw your participation at any point during the survey by closing the browser window. If you have already submitted your responses, please contact your panel provider and say you want to withdraw your consent to participate in the study. They will get in touch with us and we will exclude your data from the study. If you have any questions about our data policy, please contact predictiv@bi.team.

Please note that you cannot go back to previous pages.

Experiment page 2

Participants see one version of guidance

A
(Control)

Key messages

- If you have symptoms of coronavirus infection (COVID-19), stay at home and do not leave your house for 7 days from when your symptoms started. (See Ending Isolation section below for more information)
- This action will help protect others in your community whilst you are infectious.
- Plan ahead and ask others for help to ensure that you can successfully stay at home.
- Ask your employer, friends and family to help you to get the things you need to stay at home.
- Stay at least 2 metres (about 3 steps) away from other people in your home whenever possible.
- Sleep alone, if that is possible.
- Wash your hands regularly for 20 seconds, each time using soap and water.
- Stay away from vulnerable individuals such as the elderly and those with underlying health conditions as much as possible.
- If your symptoms worsen during home isolation or are no better after 7 days use NHS 111 online or call NHS 111; for a medical emergency dial 999.

B
(Simplified)

Staying at home: What you need to do (summary)

1. **Stay home.**
   Don't go to work, school, public areas, or routine appointments. Don't use public transport or taxis. Don't have visitors to your home.
2. **Practice good hygiene.**
   Wash your hands regularly for 20 seconds. Use tissues to cover coughs and sneezes, then bin them immediately and wash your hands.
3. **Stay away from other people.**
   Stay at least 2 metres (3 steps) away from other people. Eat and sleep alone, in a separate room if you can.
4. **Clean and dispose of waste.**
   Clean surfaces often using detergents and bleach. Use and wash your own separate toothbrush, utensils, dishes, and laundry.
5. **Take care of yourself.**
   You will most likely recover safely at home. Drink plenty of fluids, use paracetamol if you have pain, stay busy doing the things you like and keep in touch with friends and family.

C
(Visual aids)

Staying at home: What you need to do (summary)

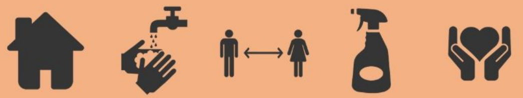

Stay at home for **7 days**

- Don't go to work, school, public areas, or routine appointments.
- Don't use public transport or taxis.
- Don't have visitors to your home.

Practice good **hygiene**

- Wash your hands regularly for 20 seconds.
- Use tissues to cover coughs and sneezes, then bin them immediately and wash your hands.

Stay away from other people

- Stay at least 2 metres (3 steps) away from other people.
- Eat and sleep alone, in a separate room if you can.

Clean and dispose of waste

- Clean surfaces often using detergents and bleach.
- Use and wash your own separate toothbrush, utensils, dishes, and laundry.

Take **care of yourself**

- Drink plenty of fluids, use paracetamol for pain, stay busy doing things you like, keep in touch with friends/family.
- Contact NHS 111 if your symptoms get worse.

Experiment page 3
Outcome measures - Key messages (9 bullets at top of doc)

PREDICTIV

Please answer the questions based on the guidance you just saw. You will earn £xx for each correct answer.

1. What should you do if you have coronavirus or symptoms of coronavirus? [Choose one answer]

- Stay at home and do not leave your house for 7 days after your symptoms started.
- Stay at home and do not leave your house for 14 days after your symptoms started.
- Visit a GP or hospital
- Tell your Local Authority that you have coronavirus
- Ring 999 and tell them you have coronavirus

2. If you have to stay at home because you have coronavirus or symptoms of coronavirus, how far away should you stay from other people in your home?

[numeric] metres or
[numeric] step
[correct = 2 metres or 3 steps]

3. For how many seconds should you wash your hands with soap and water?

[numeric] seconds - [correct = 20]

4. If you have to stay at home because you have coronavirus or symptoms of coronavirus, and then you become more sick and need medical help or advice, what should you do? [Choose one answer]

- Contact NHS 111, or 999 in an emergency
- Visit a GP, or for an emergency go to a hospital
- Search online for more information
- Ask someone to come to your house to help you
- Arrange an appointment with your GP

Experiment page 3

Outcome measures - Key messages (9 bullets at top of doc)

5. If you are staying at home because you have coronavirus or symptoms of coronavirus, which of these things should you do? Tick all that apply

- Ask other people to help you get things you need
- Have food and groceries delivered to you
- Use disinfectant or household cleaner to regularly clean surfaces
- Stay away from other people, especially older people
- Contact NHS 111 to help you get the things you need to stay at home
- Wash your towels and bedsheets everyday
- Go to the pharmacy to get medicine if you are in pain
- Allow a maximum of three visitors to your home at one time

Experiment page 3

Outcome measures - Recall of non-key messages

6. Which of the following were listed as symptoms of coronavirus? Tick all that apply.

- High temperature
- New continuous cough
- Blocked or runny nose
- Sore throat
- Muscle aches
- Sneezing
- Headache
- Pressure in your ears and face
- Loss of taste and smell
- Shortness of breath

Experiment page 3

Outcome measures - Behavioural intent

7. Would you stay at home for <u>at least 7 days</u> after the start of your symptoms if you had coronavirus or symptoms of coronavirus?

- I definitely **would** stay home for at least 7 days
- I would try to stay home for at least 7 days
- I definitely **would not** stay home for at least 7 days
- Not sure

Experiment page 3
Outcome measures - Self-efficacy & Worry

PREDICTIV

8. Looking at the guidance again, would you say it
- is easy to understand
- is confusing
- is reassuring
- makes you feel anxious
- makes you feel you would know what to do if you had coronavirus
- makes you feel the government is taking coronavirus seriously
[Answer scale = (not at all / a little / somewhat / very / extremely)]

9. **Do you have any other feedback about the guidance?**
[free text box]

Participants can see the guidance again while answering this question

Experiment page 4
Demographics / feedback

[We will also capture age, gender, income, location, rural/urban, education, smoking status, parental status].

This is the end of the survey. Thanks for participating!

If you have any feedback, please enter it in the box below.
[free text box]

Please click 'submit' below to return back to the panel website.

Additional demographics (1)

[parental_status_standard] **Please choose the options that best describe your household:**

- I am pregnant/expecting a child within the next 9 months
- I have one or more children under the age of 18 living in my household
- I have one or more children aged 18 or older living in my household
- I have no children living in my household and I am not pregnant/expecting a child within the next 9 months

[Number of people in household] **How many people live in your household including yourself?**

- 1 person
- 2 persons
- 3 persons
- 4 persons
- 5 persons
- More than 5 persons
- Prefer not to answer

Additional demographics (2)

(Age of other people in household) Excluding yourself, is anyone in your household within the following age categories? Please tick all that apply.

- 0 - 4
- 5 - 10
- 11 - 19
- 20 - 29
- 30 - 39
- 40 - 49
- 50 - 59
- 60 - 69
- 70 - 79
- 80 and above
